# In vivo conversion of rat astrocytes into neuronal cells through neural stem cells in injured spinal cord with a single zinc-finger transcription factor

**DOI:** 10.1186/s13287-019-1448-x

**Published:** 2019-12-16

**Authors:** Masoumeh Zarei-Kheirabadi, Mahdi Hesaraki, Sahar Kiani, Hossein Baharvand

**Affiliations:** 1 Department of Brain and Cognitive Sciences, Cell Science Research Center, Royan Institute for Stem Cell Biology and Technology, ACECR, Tehran, 1665659911 Iran; 2 Department of Stem Cells and Developmental Biology, Cell Science Research Center, Royan Institute for Stem Cell Biology and Technology, ACECR, Tehran, 1665659911 Iran; 3 Department of Developmental Biology, University of Science and Culture, Tehran, 1461968151 Iran

**Keywords:** Adult astrocyte, Conversion, Reprogramming, Neural stem cells, *Zfp521*, In vivo reprogramming, Spinal cord injury

## Abstract

**Background:**

Spinal cord injury (SCI) results in glial scar formation and irreversible neuronal loss, which finally leads to functional impairments and long-term disability. Our previous studies have demonstrated that the ectopic expression of *Zfp521* reprograms fibroblasts and astrocytes into induced neural stem cells (iNSCs). However, it remains unclear whether treatment with *Zfp521* also affects endogenous astrocytes, thus promoting further functional recovery following SCI.

**Methods:**

Rat astrocytes were transdifferentiated into neural stem cells in vitro by *ZFP521* or *Sox2*. Then, *ZFP521* was applied to the spinal cord injury site of a rat. Transduction, real-time PCR, immunohistofluorescence, and function assessments were performed at 6 weeks post-transduction to evaluate improvement and in vivo lineage reprogramming of astrocytes.

**Results:**

Here, we show that *Zfp521* is more efficient in reprogramming cultured astrocytes compared with *Sox2.* In the injured spinal cord of an adult rat, resident astrocytes can be reprogrammed into neurons through a progenitor stage by *Zfp521*. Importantly, this treatment improves the functional abilities of the rats as evaluated by the Basso, Beattie, and Bresnahan (BBB) locomotor rating scale and further by calculation of its subscores. There was enhanced locomotor activity in the hind limbs, step length, toe spread, foot length, and paw area. In addition, motor evoked potential recordings demonstrated the functional integrity of the spinal cord.

**Conclusions:**

These results have indicated that the generation of iNSCs or neurons from endogenous astrocytes by in situ reprogramming might be a potential strategy for SCI repair.

**Graphical abstract:**

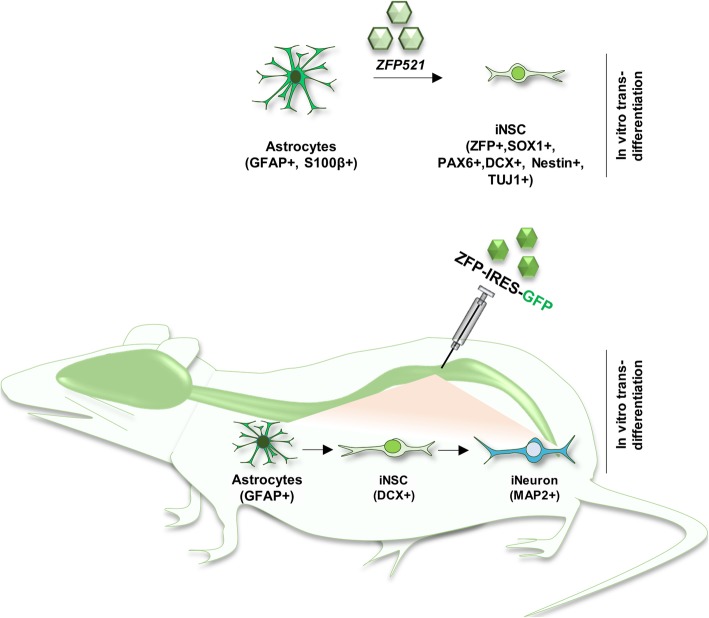

## Introduction

Spinal cord injury (SCI) is a devastating condition that leads to severe motor, sensory, and autonomic dysfunction below the point of injury [1]. Injury to the spinal cord frequently disrupts the long ascending and descending spinal tracts and results in functional impairments and long-term disability. There is currently no effective treatment for these injuries.

In order to improve recovery following SCI, different strategies under consideration include providing tissue protection, modulating circuit reorganization, or regulating neural bridging connectivity across lesions (for review see [2, 3]). Another method is the cell-grafting approach that takes advantage of neurotrophic factor-releasing cells [4], autologous engraftment of pieces of peripheral nerve [5, 6], Schwann cells [7, 8], olfactory ensheathing cells [9], oligodendrogenic neural progenitor cells [10], mesenchymal stem cells [11–13], and neural stem cells (NSCs) [14–16]. A major hurdle faced by the cell transplantation strategy is the induction of a secondary injury to the spinal cord due to the relatively large quantity of cells needed for transplantation.

In situ reprogramming or transdifferentiation of endogenous non-neuronal cells, such as astrocytes in the adult spinal cord into NSCs or neurons, represents a novel approach towards understanding and treating neural injury and degeneration. Several studies have reported the reprogramming of scar-forming astrocytes into oligodendrocytes, induced NSCs (iNSCs), and/or neurons by the forced expressions of a few transcription factors [17–25]. This strategy would likely generate a higher number of functional reprogrammed cells due to the availability of essential factors provided by the natural niche.

Reactive astrocytes recruited to the SCI-induced lesion progressively form an astrocyte “scar,” which is a physical barrier, and express molecules inhibitory to axon growth. However, a limited number of axons may pass this barrier [26, 27]. Reactive astrocytes are typically characterized by increased amounts of the intermediate filament glial fibrillary acidic protein (Gfap). Early removal of the astrocyte scar has been found to increase the size of the lesion area and reduce functional recovery in mice [26, 28]. Therefore, endogenous astrocytes might be ideal targets for in vivo lineage reprogramming. Although the reprogramming of brain or spinal cord astrocytes into neurons or neuroblasts has been previously reported [20, 21, 29, 30], there is no report of improvement in function following SCI.

Recently, we demonstrated that a single zinc-finger transcription factor, *Zfp521*, is sufficient for direct conversion of human and mouse fetal- and neonatal-derived fibroblasts into proliferating and multipotent iNSCs [31]. Our recent study indicated that mouse brain astrocytes could be reprogrammed in vitro to iNSCs by *Zfp521* [32]. Since reprogramming efficiencies of ectopic transcription factors may differ in vivo, it is unclear whether *Zfp521* can convert the fate of astrocytes in the adult rat spinal cord and improve function following SCI.

In this study, we compared the effects of *Zfp521* and *Sox2* on the generation of iNSCs in adult rat astrocytes in vitro and found that ectopic *Zfp521* was sufficient to convert astrocytes to proliferative iNSCs with an increased reprogramming efficiency compared with *Sox2* induction. We also assessed whether neurogenesis could be induced in our SCI model and found that resident astrocytes in the injured adult spinal cord could be manipulated by *Zfp521* to eventually generate mature neurons. More importantly, this transdifferentiation approach resulted in improved function following SCI and indicated its therapeutic potential.

## Materials and methods

### Isolation of rat astrocytes

All animals experiments performed in this study were in strict accordance with the guidelines of the Royan Institute Review Board and Ethics Committee. Astrocytes were extracted from the brain of an adult male Wistar rat (2–3 months old, 200–250 g). The animals were sacrificed and we extracted the cells from the gray matter of the cerebral cortexes. The gray matter was dissected and mechanically and enzymatically dissociated. Each cortical hemisphere was cut into small pieces and placed in 0.5% trypsin (Invitrogen) in Hank’s balanced salt solution (HBSS), mixed and incubated for 20–30 min at 37 °C. The suspension was filtered through a 100-μM cell strainer and centrifuged. The mixed isolated cells were cultured in astrocyte culture medium (DMEM-F12, Invitrogen) supplemented with 10% fetal bovine serum (FBS, Gibco). The cells were agitated on day 7 for 3 h at 240 rpm and overnight on day 14 at 180 rpm to remove other cell types. After 2–3 passages, the culture was purified and analyzed.

### Lentivirus preparation

Lentivirus was used to deliver the factors into the adult rat-derived astrocytes and injured spinal cord parenchyma. For the preparation of these particles, human embryonic kidney (HEK 293T) cells were transfected with *TRE-promoter*-*Zfp521*, *TRE-promoter*-*Sox2*, *pSFFV-IRES-GFP*, the *Zfp-IRES-GFP* construct, and packaging plasmids (pCMV-vsvg and pCMV-gp) using Lipofectamine 3000 (Life Technologies). The medium from the transfected cells was collected and concentrated by ultracentrifugation (20,000 rpm for 2 h at 4 °C).

### Transduction of adult rat-derived astrocytes

Adult rat brain-derived astrocytes were seeded onto tissue culture dishes coated with 0.001% poly-l-ornithine (Sigma-Aldrich, P4707) and 10 mg/ml laminin (Sigma-Aldrich, L2020). Extracted cells were induced with inducible lentiviral vectors that expressed the mouse *Zfp521* or *Sox2* (*TRE-promoter*-*ZFP521* and *TRE-promoter*-*Sox2* (Life Technologies)) and cultured in astrocyte medium (AM). The culture medium was replaced with induction medium (IM) 2 days after viral transduction. This medium included DMEM high glucose supplemented with 10% knockout serum replacement, 1% non-essential amino acids, 2 mM l-glutamine, ITS (1 mg/ml insulin, 0.55 mg/ml transferrin, and 0.67 mg/ml selenium), 1% N2, 0.05% B27, and 1% penicillin/streptomycin (all from Invitrogen). For the induction of ectopic expression, doxycycline (Dox, 2 mg/ml) was added to the IM. The medium was changed every 2 days. Epidermal growth factor (EGF, 20 ng/ml; Royan Biotech) and basic fibroblast growth factor (bFGF, 80 ng/ml; Royan Biotech) were gradually added to the IM. After 3 weeks, Dox was removed and the induction medium was replaced with neural stem cell medium (NSCM) supplemented with EGF and bFGF.

When cultured cells showed 60–70% confluency, they were trypsinized and transferred into poly-l-ornithine/laminin-coated 6-cm dishes in NSCM. Cells at this stage were considered to be at passage 1. The medium was changed every 2 days.

The growth curve was calculated by counting 500 × 10^3^ cells in 6-cm plates. Cells were passaged once they reached a density of 80–90% confluency, once every 3–4 days, at a split ratio of 1:2. iNSCs were passaged for ten serial passages of five replicates per passage.

We evaluated the multipotency of these iNSCs by culturing them in NSCM without EGF and bFGF.

### Cellular senescence activity assay

Cellular senescence was examined by measuring the activity of acidic senescence-associated β-galactosidase (SA-β-Gal) using a Cellular Senescence Activity Assay kit (Enzo Life Sciences) following the vendor’s protocol [33]. iNSCs at passages 3 and 10 were assessed.

### Contusion spinal cord injury modeling

Adult male wild-type Wistar rats (200–250 g) were used in this study. The rats were anesthetized with intraperitoneal injections of ketamine (100 mg/kg) and xylazine (10 mg/kg). A 3 cm × 6 cm area was then longitudinally shaved, the exposed skin was cleaned, and an approximately 4 cm incision was made using a #10 blade centered on the T10 mark. Laminectomy was performed to expose the spinal cord at the T9–11 level. The vertebral column was fixed by two clamps and the contusion was made using an NYU impactor (10 g, 25 mm). After surgery, the rats were given an antibiotic (Enrofeloxacine, 5 mg/kg) for 1 week. Their bladders were manually expressed two times per day until the return of bladder function. The rats were monitored for 1 week for the presence of any infection (blood in urine, whitish color, or foul odor), decreased physical activity, or problems with wound healing. Infections were controlled with an increased dosage of antibiotics.

### Injection of *pSFFV-IRES-GFP* and *Zfp-IRES-GFP* lentiviruses and astrocytes in the injured spinal cord

Using a Hamilton syringe and a 30-gauge 45° beveled needle (Hamilton, Reno, NV), we injected 3 μl of the *pSFFV-IRES-GFP* and *Zfp-IRES-GFP* lentiviruses into the spinal cord parenchyma, 2 mm rostral to the lesion site, at 1 week post-injury. The virus was slowly injected over a 5-min period, after which the needle was held in place for an additional 2 min and then slowly withdrawn. In the AST-Zfp group, 2 × 10^5^ *Zfp521*-transfected astrocytes and the same count of mock-transfected astrocytes in the AST-Mock group were transplanted into the spinal cord parenchyma at 2 mm rostral to the lesion site 1 week post-injury.

### Immunocytochemistry and immunohistofluorescence analysis

Cells were fixed in 4% paraformaldehyde (Sigma-Aldrich, P6148) for 15 min. Permeabilization of cells was performed using 0.5% Triton (Sigma-Aldrich, T8532), and the cells were blocked in serum obtained from the host of the secondary antibody for 1 h. Cells were incubated with primary antibodies overnight at 4 °C and with secondary antibodies for 1 h (Alexa Fluor, Invitrogen). Additional file 4: Table S1 lists the antibodies used in this analysis.

The animals were perfused with 4% (w/v) paraformaldehyde in phosphate-buffered saline. Spinal cords were surgically removed, post-fixed overnight in 4% (w/v) paraformaldehyde, and cryoprotected with 30% sucrose at 4 °C for 48 h. We used a cryostat to cut 10-mm-thick transverse or longitudinal sections of spinal cords that spanned the injury sites. Then, permeabilization of tissues was performed using 0.5% Triton (Sigma-Aldrich, T8532), and the samples were blocked in serum obtained from the host of the secondary antibody for 1 h. The samples were incubated with primary antibodies overnight at 4 °C and with secondary antibodies for 1 h (Alexa Fluor, Invitrogen). Nuclei were counterstained with 4′,6-diamidino-2-phenylindole (DAPI). Additional file 4: Table S1 lists the antibodies used in this analysis. An Olympus IX71 fluorescence microscope with a DP72 digital camera and analySIS LS Starter software (version 3.2) was used to capture and analyze all images. Cell counts were performed using ImageJ software.

### RNA isolation, reverse transcription, and quantification

Total RNA was prepared using TRIzol reagent (Invitrogen) according to the manufacturer’s recommendations. A UV/visible spectrophotometer (WPA, Biowave II) was used to determine the concentration and purity of the RNA obtained. Subsequently, 2 μg of total RNA was reverse transcribed to the first-strand cDNA using a Revert Aid First-strand cDNA Synthesis Kit (K1622, Thermo Scientific) in a 20 μl reaction, according to the manufacturer’s instructions. cDNA was diluted at a ratio of 1:5. A total of 2 μl of cDNA was used for quantitative real-time PCR (qRT-PCR) in a 20 μl PCR reaction that contained 10 μl 2x Power SYBR Green Master Mix (Applied Biosystems) and 1 μl of 5 pmole forward and reverse primers. Reactions were run on a Rotor Gene 6000 (Corbett Life Science). All qRT-PCR experiments were performed using three technical and independent biological replicates. Data were normalized against *GAPDH* and presented relative to the expression of each indicated gene in astrocytes. Additional file 4: Table S2 lists the primer sequences used in this study.

### Basso, Beattie, and Bresnahan (BBB) locomotor rating scale

The rats’ locomotion was scored weekly using the Basso, Beattie, and Bresnahan (BBB) locomotor rating scale [34]. The scale (0–21) represents sequential recovery stages and categorizes combinations of rat joint movement, hind limb movement, step, forelimb and hind limb coordination, trunk position and stability, paw placement, and tail position. To calculate BBB subscores, individual categories of BBB outcomes were quantified as previously indicated [35]. Each category within the subscores indicates that independently specific aspects of locomotion were altered by treatment.

### Footprints

The locomotor activity of the hind limbs was measured 6 weeks post-transduction (WPT) using footprints. Two dyes were applied to the soles of the hind paws, and the rats were allowed to walk on a narrow white paper-covered corridor [36]. At least 4–5 sequential steps were used to determine the mean values for each measurement, including stride angle, stride length, foot length, step width, toe spread, and paw area [34]. The measurement was made using ImageJ software.

### Motor-evoked potential (MEP) assay

One week before the SCI surgery, the rats were anesthetized with intraperitoneal injections of ketamine (100 mg/kg) and xylazine (10 mg/kg). Their heads were fixed in a stereotaxic device (Stoelting, USA). An incision was made along the midline of each skull, and two holes were drilled in the left hemisphere using a standard dental drill (Faro, Italy). The first hole was located 2 mm anterior to the bregma and 2 mm lateral to the midline (motor cortex, evoked electrode). The second hole was located 6 mm posterior to the bregma and 4 mm lateral to the midline (reference electrode). For transcranial electrical stimulation of the motor cortex, screw electrodes were implanted at a depth of 0.75 mm to ensure contact with the cortex and to avoid placement of any pressure on the dura mater. Carboxylate dental cement (Acropars, Iran) was used to fix the electrodes.

Five days after electrode implantation, baseline recordings were performed. Recording needle electrodes were inserted intramuscularly into the middle of the tibial anterior muscle of the right hind limb of each rat. Reference needle electrodes were inserted into the ankle of the same muscle. A footpad was attached as the ground electrode. Stimulation and recordings were performed using an electro-module device and recording software (R12, ScienceBeam, Iran). The motor cortex was stimulated using 5 pulses of 1 mA intensity, 200 μs duration, and 15 Hz frequency. This stimulation pattern was repeated 15 times with 70 ms intervals. The maximum peak attained in the wave pattern was considered for amplitude, and the mean amplitude and latency were reported for three randomly selected waves. MEP recordings were obtained 2 days before SCI (intact), 2 days after SCI, and six WPT.

### Statistical analysis

All data are presented as mean ± SD. Statistical differences were evaluated by ANOVA and the Mann-Whitney *U* test, Tukey’s post hoc test, or the unpaired *t* test. *p* ≤ 0.05 was considered statistically significant.

## Results

### Establishment of expandable induced neural stem cells (iNSCs) from adult rat astrocytes in vitro

Extracted astrocytes expressed astrocyte markers S100β (a calcium-binding protein in astrocytes) and Gfap, but not neuronal and/or NSC markers Sox2, Nestin, Doublecortin (Dcx; a microtubule-associated protein that is broadly expressed in neuroblasts and immature neurons), and β-tubulin III (Tuj1; a pan-neuronal marker) at the RNA and protein levels (Additional file 1: Figure S1).

**Fig. 1:**
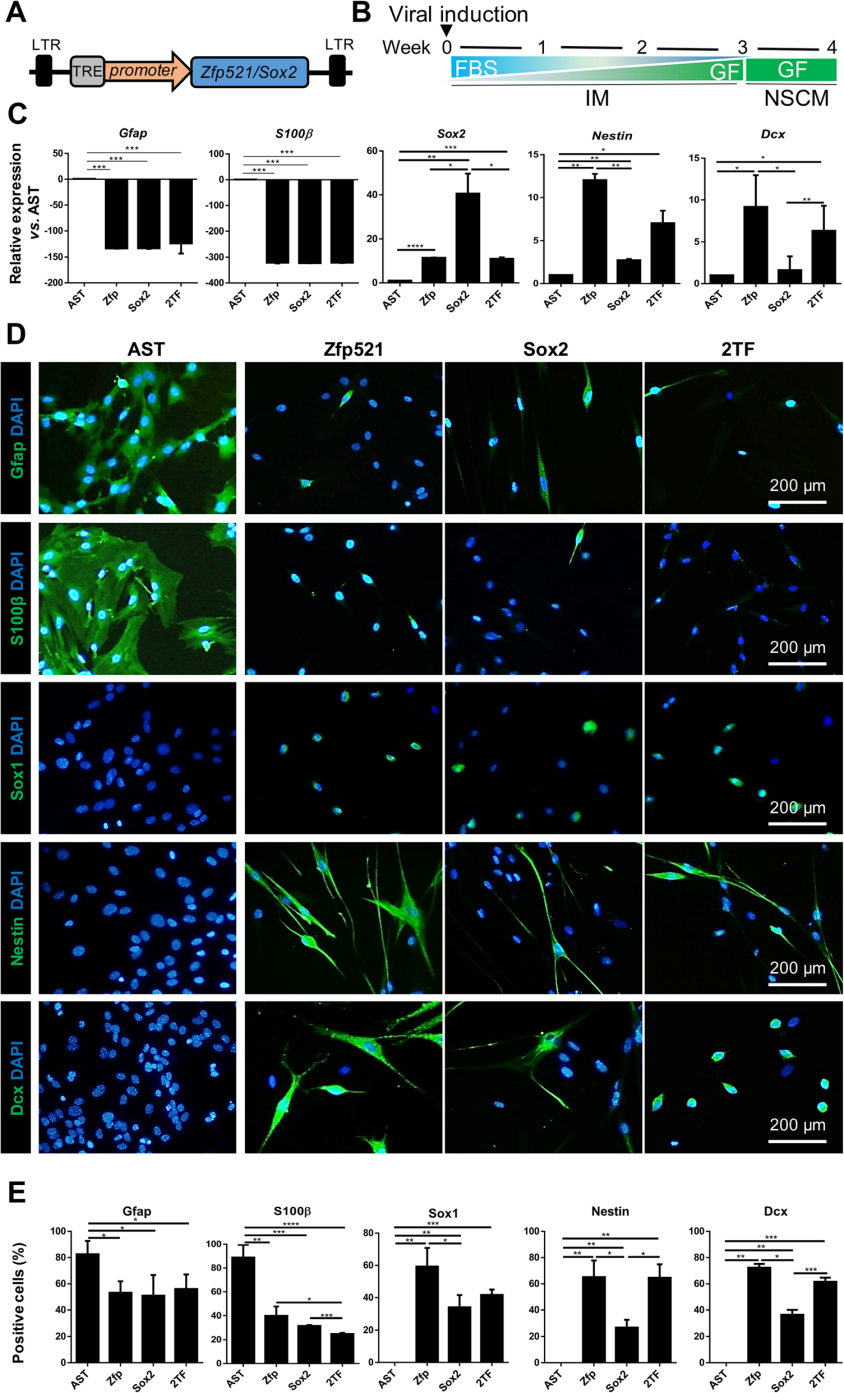
Reprogramming adult rat derived astrocytes. **a** A map of the lentiviral vectors, *Sox2* and *Zfp521*. The vector is inducible by doxycycline (Dox) and contains the TRE element, a promoter, and *Zfp521* or *Sox2* CDS. **b** A summary of the experimental design for transdifferentiation of astrocytes into induced neural stem cells (iNSCs). Viral induction was performed at week 0. The cells were cultured in induction medium (IM) in the presence of Dox for 3 weeks with decreasing concentrations of fetal bovine serum (FBS) and increasing concentrations of growth factors (GF), bFGF, and EGF. The culture medium was then changed to neural stem cell medium (NSCM) supplemented with GF until week 4. The cells were transduced with *Zfp521* (Zfp), *Sox2*, or both transcription factors (2TF). **c** qRT-PCR analysis of astrocyte (AST) markers (*Gfap*, *S100β*) and NSC markers (*Sox2*, *Nestin*, and *Dcx*) at 4 WPT. Data were normalized against *GAPDH* and presented relative to the expression of each indicated gene in astrocytes. **d** Immunofluorescent staining of cells for astrocytes and iNSC at 4 weeks post-transduction (WPT). Nuclei were counterstained with DAPI. **e** Quantification of protein markers revealed by immunostaining in **d**. Data in **c** and **e** are given as mean ± SD for three independent experiments. Data were analyzed by ANOVA and Tukey test as post hoc. *p < 0.05, **p < 0.01, and ***p < 0.001. The number of counted cells is presented in Additional file 4: Table S3

In order to assess the ability of *Zfp521* to reprogram adult rat astrocytes into iNSCs, we overexpressed *Zfp521*, *Sox2*, or the combination of both (2F) using a lentiviral delivery system (Fig. 1a). For controlled ectopic expression of the transgene, Dox-inducible lentiviruses were used. *Sox2* was used as a control reprogramming factor because it has been used for both spinal cord- and brain-derived astrocytes [20, 29]. The cells were cultured in IM in the presence of Dox for 3 weeks and then in NSCM (Fig. 1b).

Overexpressions of *Zfp521*, *Sox2*, or their combination (2F) in rat astrocytes resulted in the down-regulation of the astrocyte markers *S100β* and *Gfap* and the up-regulation of NSC markers *Sox2*, *Nestin*, and *Dcx* at the RNA level as quantified at week 4 in vitro (Fig. 1c). Immunostaining results confirmed down-regulation of the astrocyte markers and up-regulation of the NSC markers (Fig. 1d, e). Transduction with an empty vector (astrocytes + empty vector in astrocyte medium [A] and astrocytes + empty vector in induction medium and DOX [AD]) did not result in up-regulation of the NSC markers Nestin and Dox (Additional file 2: Figure S2). A loaded vector in the absence of DOX (astrocytes + *Zfp521* without DOX [AZ] or astrocytes + *Zfp521* with DOX [AZD]) also did not lead to up-regulation of the NSC markers (Additional file 2: Figure S2).

A comparison of gene expression in the reprogrammed cells showed more up-regulation of Nestin and Dcx at the RNA and protein level when *Zfp521* was overexpressed compared with *Sox2* or 2TF overexpression (Fig. 1c, e)*.* Therefore, we continued our experiments with only *ZFP521.*

**Fig. 2:**
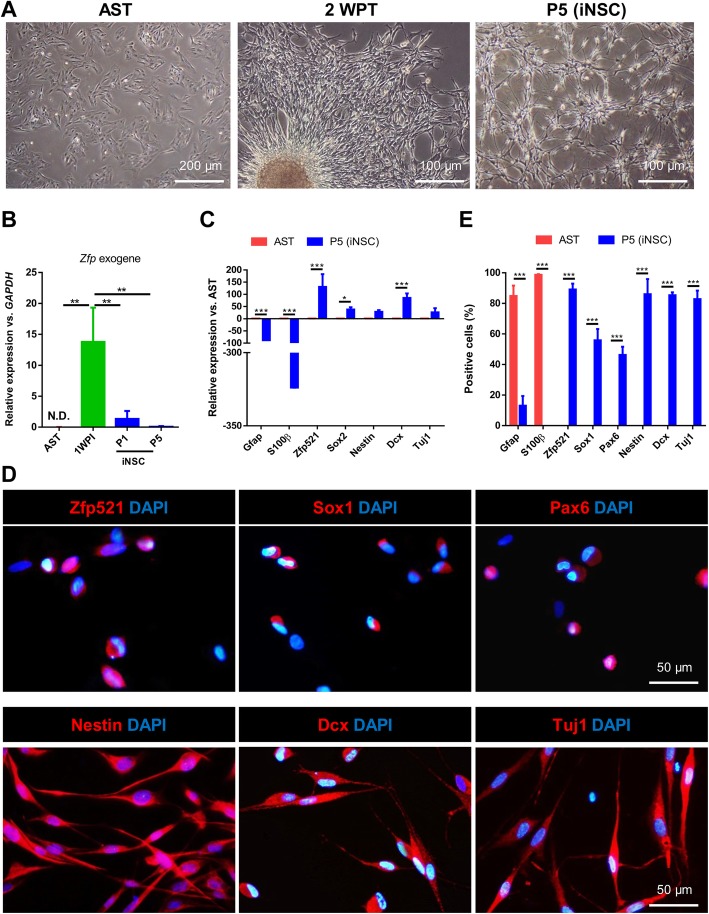
Generation and characterization of induced neural stem cells (iNSCs) derived from adult rat astrocytes. **a** Phase-contrast images of astrocytes before induction and at 2 weeks post-transduction (WPT), induced neural stem cells (iNSCs) at passage 5 (P5). **b** qRT-PCR analysis of exogen *Zfp521* shows decreasing expression levels with subsequent passages. **c** qRT-PCR analysis of astrocytes (AST) and NSC markers at P5. Data were normalized against *GAPDH* and presented relative to the expression of each indicated gene in astrocytes. **d** Immunofluorescent staining of iNSC at P5. Nuclei were counterstained with DAPI. **e** Quantification of protein markers revealed by immunostaining in **d**. Data in **b**, **c**, and **e** are given as mean ± SD of three independent experiments. Data were analyzed by ANOVA and Tukey test as post hoc. *p < 0.05, **p < 0.01, and ***p < 0.001. The number of counted cells is presented in Additional file 4: Table S3

Two weeks after *Zfp521* transduction, several cell spheroids emerged. These spheroids were morphologically similar to spheres formed by wild-type NSCs (Fig. 2a).

The efficiency of reprogramming was estimated using the number of Dcx^+^ cells 4 weeks after transduction relative to the number of astrocytes that had been initially seeded in three independent experiments. The efficiency of reprogramming for adult rat-derived astrocytes with *Zfp521* was 46.10 ± 2.9%. iNSCs generated by this method could be expanded onto laminin/poly-l-ornithine-coated dishes and showed morphological homogeneity (Fig. 2a).

qRT-PCR indicated that exogenous *Zfp521* was not expressed in the absence of Dox (Fig. 2b) and the expression of astrocyte markers, *S100β* and *Gfap*, were down-regulated while *Sox2*, *Nestin*, *Dcx*, and *Tuj1* were up-regulated at passage 5 in iNSCs (Fig. 2c). In addition, immunofluorescence analyses showed that iNSCs expressed endogenous Zfp521, Sox1, Pax6, Nestin, Dcx, and Tuj1 markers while astrocyte markers S100β and Gfap were down-regulated (Fig. 2d, e).

**Fig. 3:**
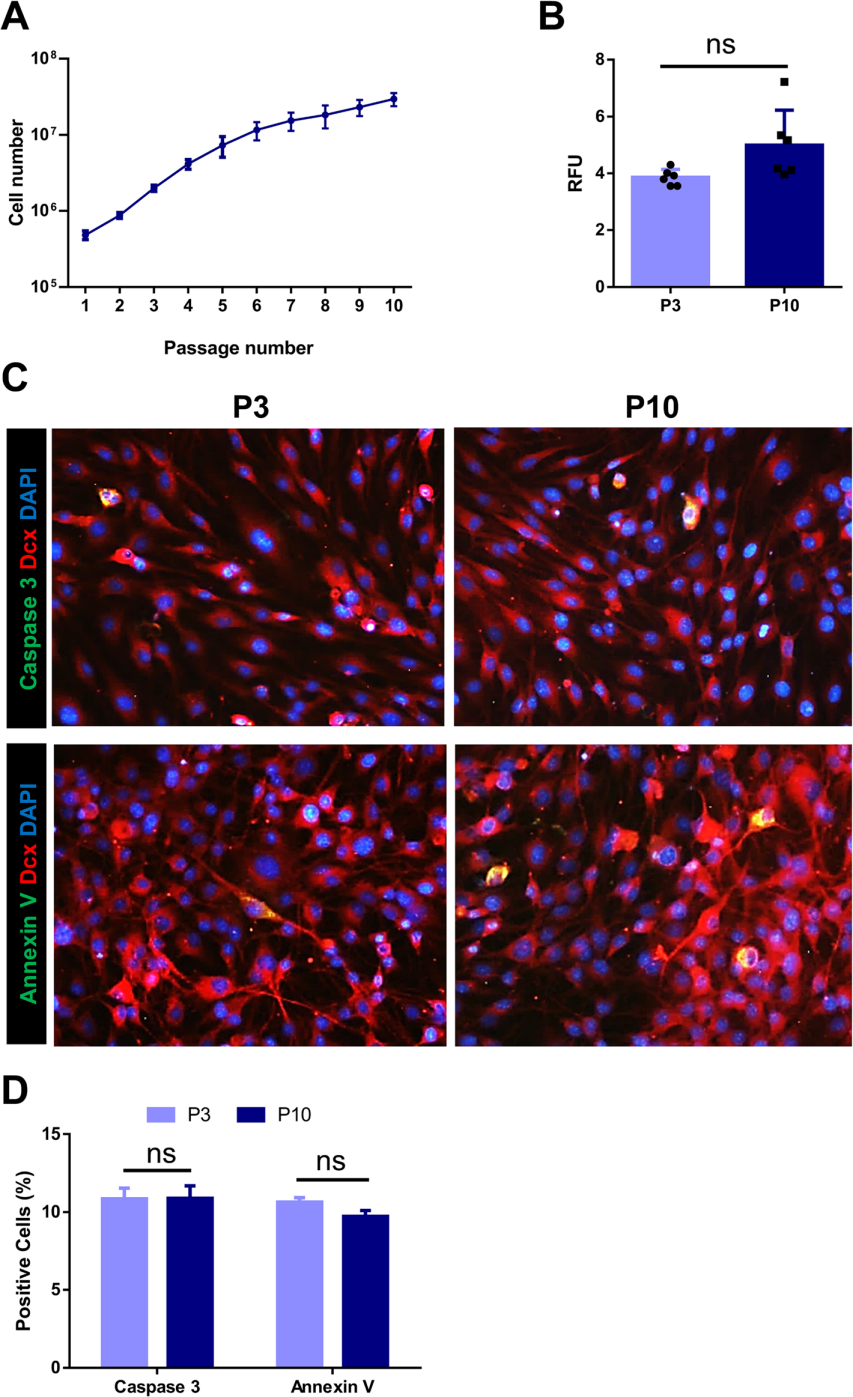
The assessment of apoptosis in adult astrocyte-derived induced neural stem cells (iNSCs). **a** A growth curve of induced neural stem cells (iNSCs) during different passages. **b** SA-β-Gal activity in in iNSCs after 3 and 10 passages. **c** Immunostaining of iNSCs for Caspase 3 and Annexin V after 10 passages. **d** Quantification of the percentage of Caspase 3-positive and Annexin V-cells after 3 and 10 passages revealed by immunostaining in **c**. Data in **b** and **d** are given as mean ± SD of three independent experiments. Data were analyzed by the unpaired *t* test. The number of counted cells is presented in Additional file 4: Table S3

In order to perform additional characterization of the iNSCs, we monitored their growth curve at passage 10 (Fig. 3a). Assessment of the aging of these iNSCs by SA-β-Gal activity at passages 3 and 10 showed a non-significant difference in relative fluorescence unit (RFU) of the same number of cultured cells in both passages (Fig. 3b). We assessed apoptosis in the iNSCs by staining the cells with anti-Caspase 3 and Annexin V at passages 3 and 10. We observed no significant difference in apoptosis at both passages (Fig. 3c, d).

**Fig. 4:**
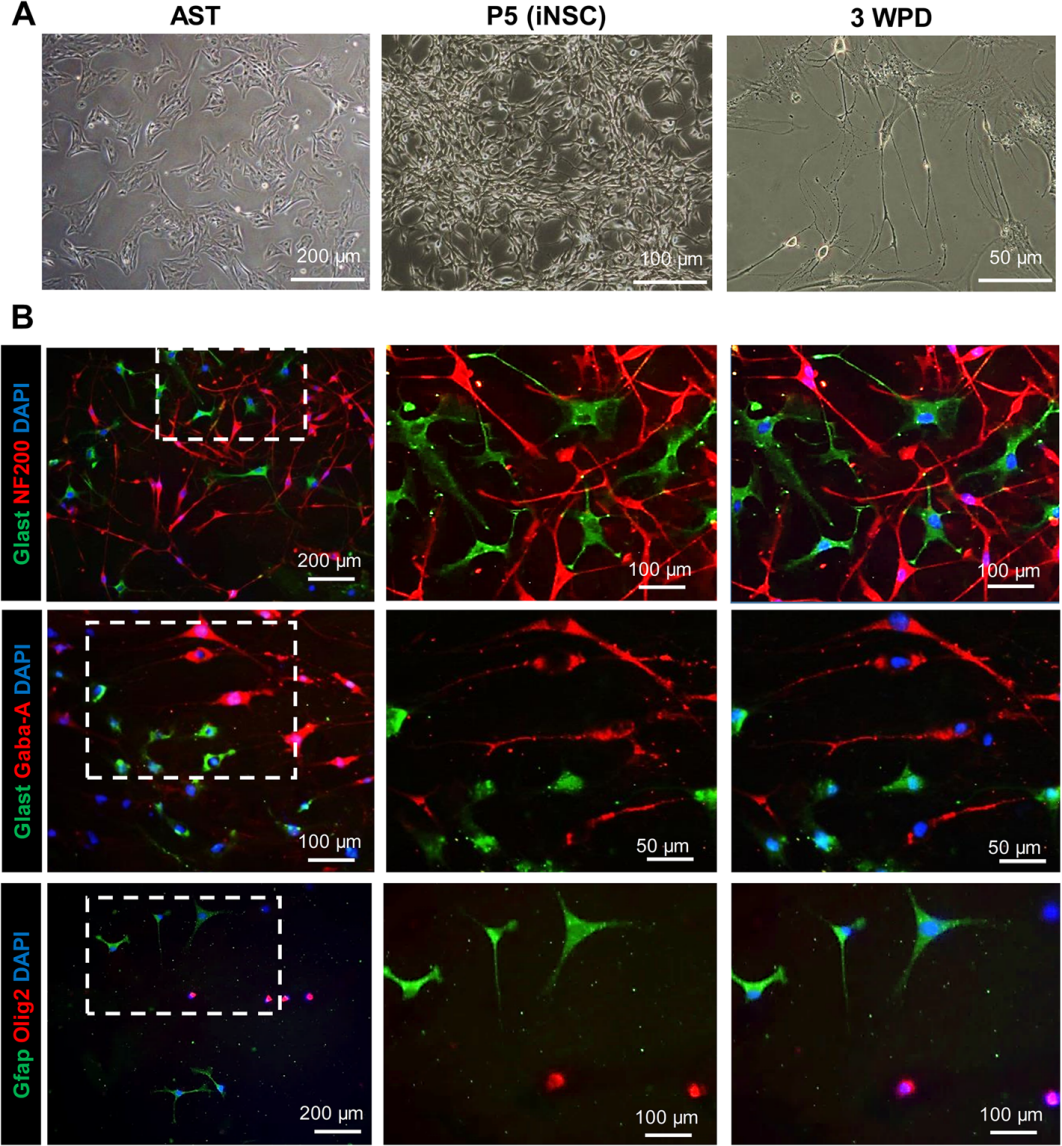
The differentiation of adult astrocyte-derived induced neural stem cells (iNSCs) in vitro. **a** Phase-contrast images of astrocytes (AST), induced neural stem cells (iNSCs) at passage 5 (P5), and 3 weeks post-differentiation (3 WPD). **b** Immunostaining of iNSCs differentiated into NF200 and Gaba-A receptor-positive neurons, Glast and glial fibrillary acidic protein (Gfap)-positive astrocytes, and Olig2-positive oligodendrocytes. Nuclei were counterstained with DAPI

iNSCs were allowed to spontaneously differentiate into NSCM without growth factors to assess their multipotency and ability to generate the three main neural cell types (neurons, astrocytes, and oligodendrocytes) (Fig. 4a). Immunostaining of the cells at 3 weeks post-differentiation (3 WPD) showed that the iNSCs could differentiate into neurons (NF200 and Gaba-A receptor), astrocytes (Glast and Gfap), and oligodendrocytes (Olig2) (Fig. 4b).

Collectively, these results demonstrated that *Zfp521* could reprogram adult rat astrocytes in vitro into stable self-renewing iNSCs.

### Functional analyses of the adult rat contusion model of spinal cord injury (SCI) after in vivo transduction with *Zfp521*

We sought to determine whether the adult rat spinal cord astrocytes could be converted into iNSCs with the same factor and whether transduced cells or exogenous *Zfp521* improved a degree of function in the rat SCI model. The SCI model was generated in the adult rat by a contusion at the T9–11 level (Additional file 3: Figure S3A) and resulted in paralysis of the hind limbs. Histological analyses of the spinal cords at 1 week post-injury showed high expression of Gfap^+^ cells around the formed cavity. Additional file 3: Figure S3B-D shows images of longitudinal and transverse sections of the injured spinal cord with hematoxylin and eosin (H&E) staining and immunostaining for Gfap. The expression of Gfap was lower in the rostral and caudal sections of the lesion site (Additional file 3: Figure S3C). Furthermore, scar tissue was detected by the expression of Gfap and fibronectin around the site of the injury (Additional file 3: Figure S3D).

**Fig. 5:**
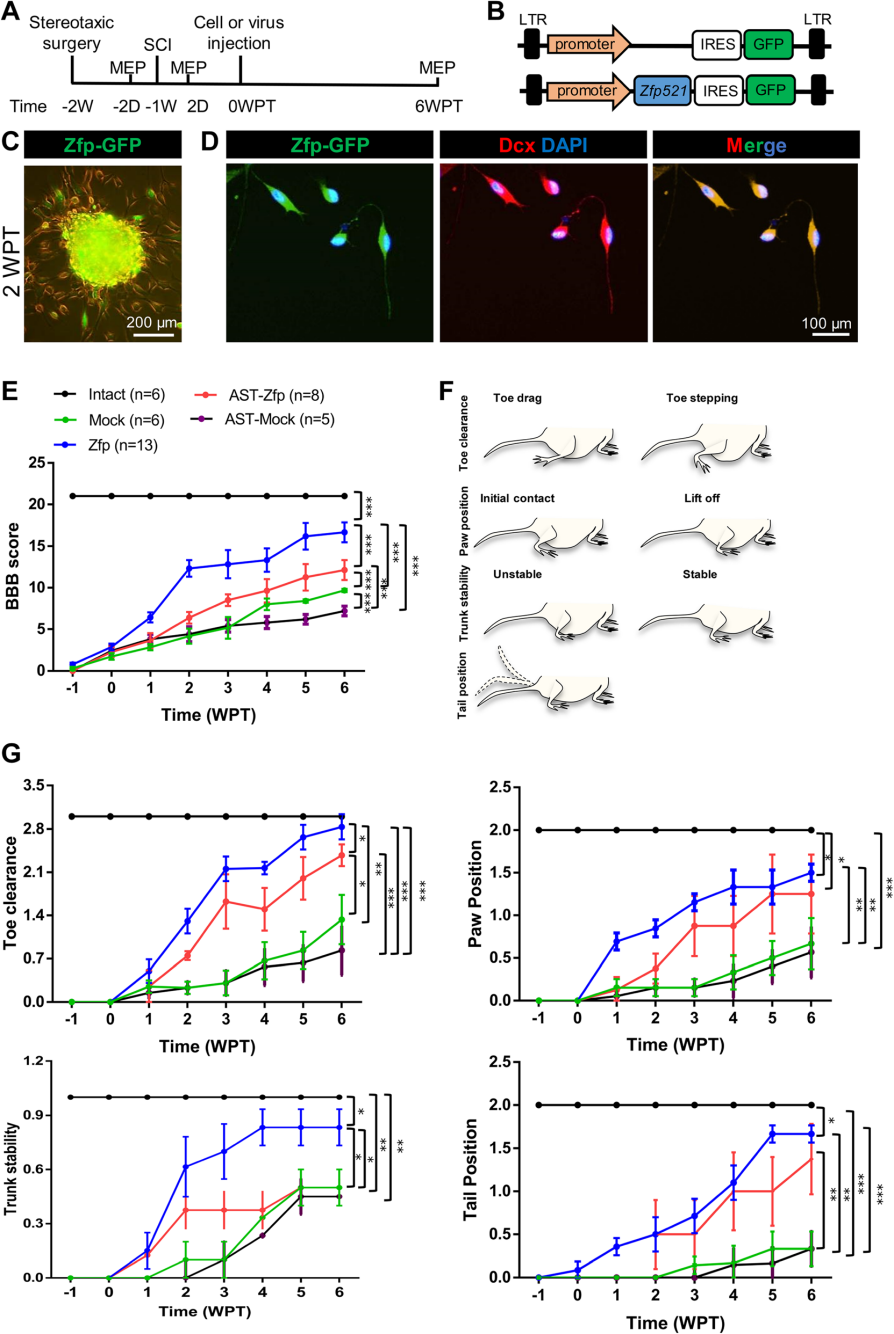
The behavioral scores of rats with spinal cord injury (SCI) after injection of Zfp-IRES-GFP (Zfp-GFP) and pSFFV-IRES-GFP (Mock) lentiviral particles at 6 weeks post-transduction (WPT). **a** Schematic of the experimental procedure used for in vivo reprogramming and analysis over 7 weeks. The stereotaxic surgery was performed 1 week before SCI. The contusion model was created at week 1, and lentiviruses that contained *Zfp-IRES-GFP* or *pSFFV-IRES-GFP* were injected at 0 weeks post-transduction (WPT). Electrophysiological examinations were performed 2 days before SCI, 2 days after SCI, and at 6 WPT by MEP. d, day; w, week; MEP, motor evoked potential; SCI, spinal cord injury. **b** *Zfp-IRES-GFP* vector structure. The vector included an SFFV promoter, a *ZFP521* CDS, and GFP that were linked by an IRES sequence. **c** Fluorescent and bright-field image of transduced astrocytes by *Zfp-GFP* vector at 1 WPT. **d** The immunofluorescent staining of transduced cells at 4 WPT for Dcx. Note that flat astrocytes were converted into Dcx^+^ bipolar cells. **e** The behavioral scores and open-field locomotor assessment as measured by the Basso, Beattie, and Bresnahan (BBB) locomotor rating scale. The animals in intact (or normal, *n* = 6), Mock (*n* = 6), Zfp (*n* = 13), AST-Mock (*n* = 5), and AST-Zfp (*n* = 8) groups were assessed weekly for 7 weeks. The BBB analysis showed a significant improvement in the AST-Zfp group in comparison with the Mock and AST-Mock group and an even greater improvement in the Zfp group. **f**, **g** BBB scores were further analyzed by calculating subscores. BBB subscores represent measures of paw position, toe clearance, trunk control, and tail position made independent of all other observable traits. Analysis of BBB subscores showed significant improvement in paw position, toe clearance, trunk control, and tail position for the Zfp group at 6 WPT. Data in **e** and **g** are given as mean ± SD. Data were analyzed by ANOVA and Mann-Whitney *U* test as post hoc. *p < 0.05, **p < 0.01, and ***p < 0.001

Four groups of rats were treated 1 week post-injury: a mock group that received the empty vector (Mock, *n* = 6); a group that received the Zfp521 vector (Zfp, *n* = 13); a group that received Zfp astrocytes (2 × 10^5^ cells, AST-Zfp, *n* = 8); and a group that received mock astrocytes (2 × 10^5^ cells, AST-Mock, *n* = 5) (Fig. 5a). We used a Zfp-IRES-GFP construct (Zfp-GFP) under the control of an SFFV promoter to trace the insertion of the lentiviral vector (Fig. 5b). Transduction of astrocytes with this vector showed the generation of green aggregates at 1 WPT (Fig. 5c). These cells also expressed Dcx at 4 WPT (Fig. 5d).

The functional abilities of the rats were analyzed weekly for 7 weeks after the injury and graded according to the BBB locomotor rating scale [34]. Data analysis revealed significant improvements in the AST-Zfp group compared with the Mock and AST-Mock groups (Fig. 5e, *p* < 0.001), and in the Zfp group compared with the AST-Zfp group at 6 WPT (Fig. 5e, *p* < 0.001). Differences were also found between the intact group and all-treated rats at 6 WPT (Fig. 5e, *p* < 0.001).

BBB scores were further analyzed by calculating the subscores [34, 35, 37–39], which allows for the characterization of the individual aspects of locomotion, either alone or in combination. Because the subscore can only quantify characteristics of locomotion that are present once the animal can take a step, this measure allows a more targeted and expanded evaluation of stepping quality than the basic BBB score [40]. Figure 5f and g show an analysis of the BBB subscores at 6 WPT. The BBB subscores represent measures of paw position, toe clearance, trunk control, and tail position, independent of all other observable traits (Fig. 5f). These BBB subscores were significantly improved in the Zfp group compared with the Mock, AST-Mock, or AST-Zfp group as these rats stepped earlier and displayed a more normal stepping pattern (*p* < 0.05, Fig. 5g).

**Fig. 6:**
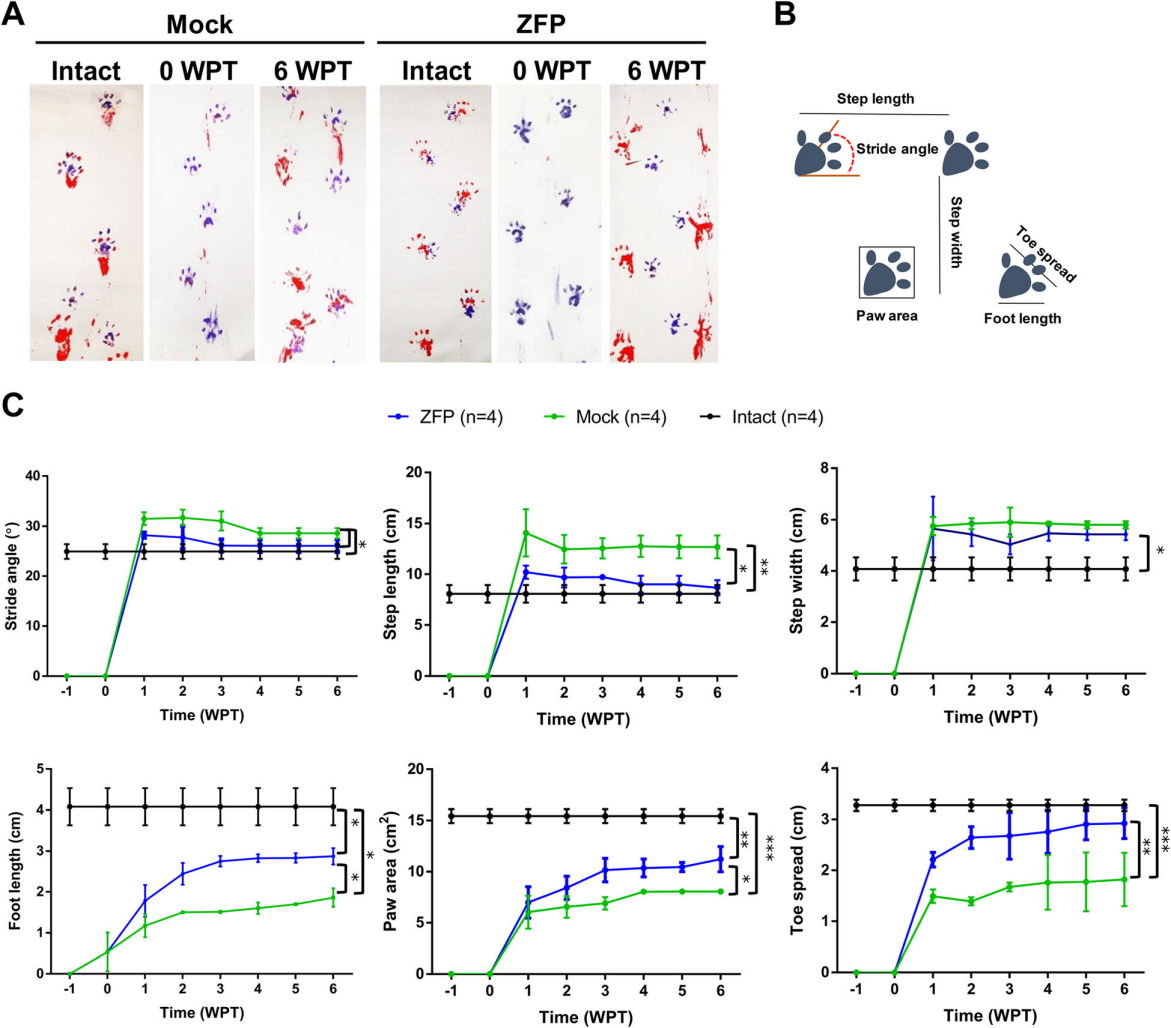
Footprint test. **a** Representative footprint analysis before spinal cord injury (SCI) (intact), 0 weeks post-transductin (WPT), and 6 WPT in the Mock and ZFP groups. These photographs show the walking pattern of rats on a narrow corridor. **b** Schematic used for quantifying stride length, step width, foot length, toe spread, and stride angle. **c** Graphs of all footprint parameters. Footprints parameters including step length, toe spread, foot length, and paw area improved significantly in the *Zfp521* group. Data are plotted as mean ± SD. *n* = 4 per group. Data were analyzed by ANOVA and Mann-Whitney *U* test as post hoc. *p < 0.05, **p < 0.01, and ***p < 0.001

We analyzed the footprint parameters at 6 WPT to assess the locomotor activity of the hind limbs (Fig. 6a, b). The stride angle, step length, and toe spread in the Zfp-treated group showed values similar to the intact group and improved in comparison with the Mock group (Fig. 6c, *p* < 0.05). The step width parameter showed no improvement in the ZFP group. The foot length and paw area parameters were improved in the Zfp group compared with the Mock group (Fig. 6c, *p* < 0.05).

**Fig. 7:**
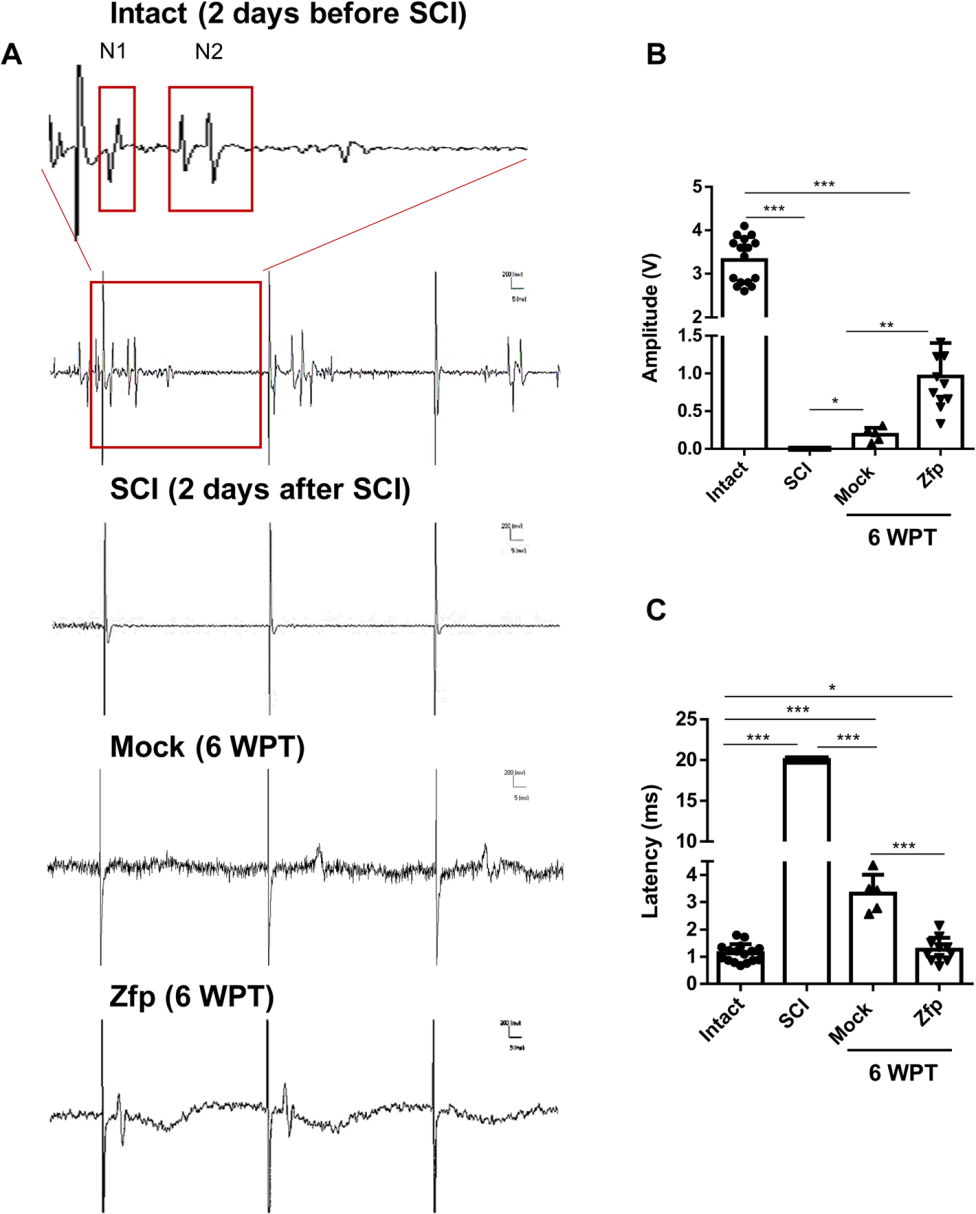
Motor-evoked potential (MEP) recordings. **a** Representative motor-evoked potential (MEP) waves obtained 2 days before spinal cord injury (SCI; *n* = 16), 2 days after SCI (*n* = 16), and at 6 weeks post-transduction (WPT) in the Mock (*n* = 5) and Zfp (*n* = 11) groups. In intact traces (2 days before SCI), two separated waves, N1 and N2, were elicited by each stimulation. Note that N1 MEPs are a single transcranial electrical pulse, while N2 MEPs are polyphasic pulses. Neither wave could be elicited 2 days after the SCI. At 6 WPT, only N1 MEPs were detected in the Mock and ZFP groups. **b** In the Zfp-treated group, the amplitude of N1 MEPs increased significantly at 6 WPT. **c** In the Zfp-treated group, the latency of N1 MEPs decreased significantly at 6 WPT. Data are shown as mean ± SD. Data were analyzed by ANOVA and the Mann-Whitney *U* test as post hoc. *p < 0.05, **p < 0.01, and ***p < 0.001

MEP recordings were performed 2 days before SCI, 2 days after SCI, and at 6 WPT to assess the functional integrity of the spinal cord (Fig. 7a). We stimulated the left motor cortex and recorded the right tibia muscle. In intact recordings (2 days before SCI), every excitation elicited two separate waves, N1 MEPs and N2 MEPs [41]. N1 MEPs were single transcranial electrical pulses that were evoked with a short latency and recorded in the tibia anterior muscles in all of the animals that we tested. In contrast, N2 MEPs are polyphasic component pulses that were evoked with a longer latency. Neither N1 nor N2 MEPs occured 2 days after SCI or appeared with a smaller amplitude (Fig. 7b). At 6 WPT, only N1 MEPs were detected in the Mock and ZFP groups. At 6 WPT, wave amplitude (Fig. 7b) increased significantly in the Zfp group and latency (Fig. 7c) decreased in comparison with the Mock group.

### In vivo reprogramming of astrocytes in the adult contusion model of spinal cord injury (SCI)

**Fig. 8:**
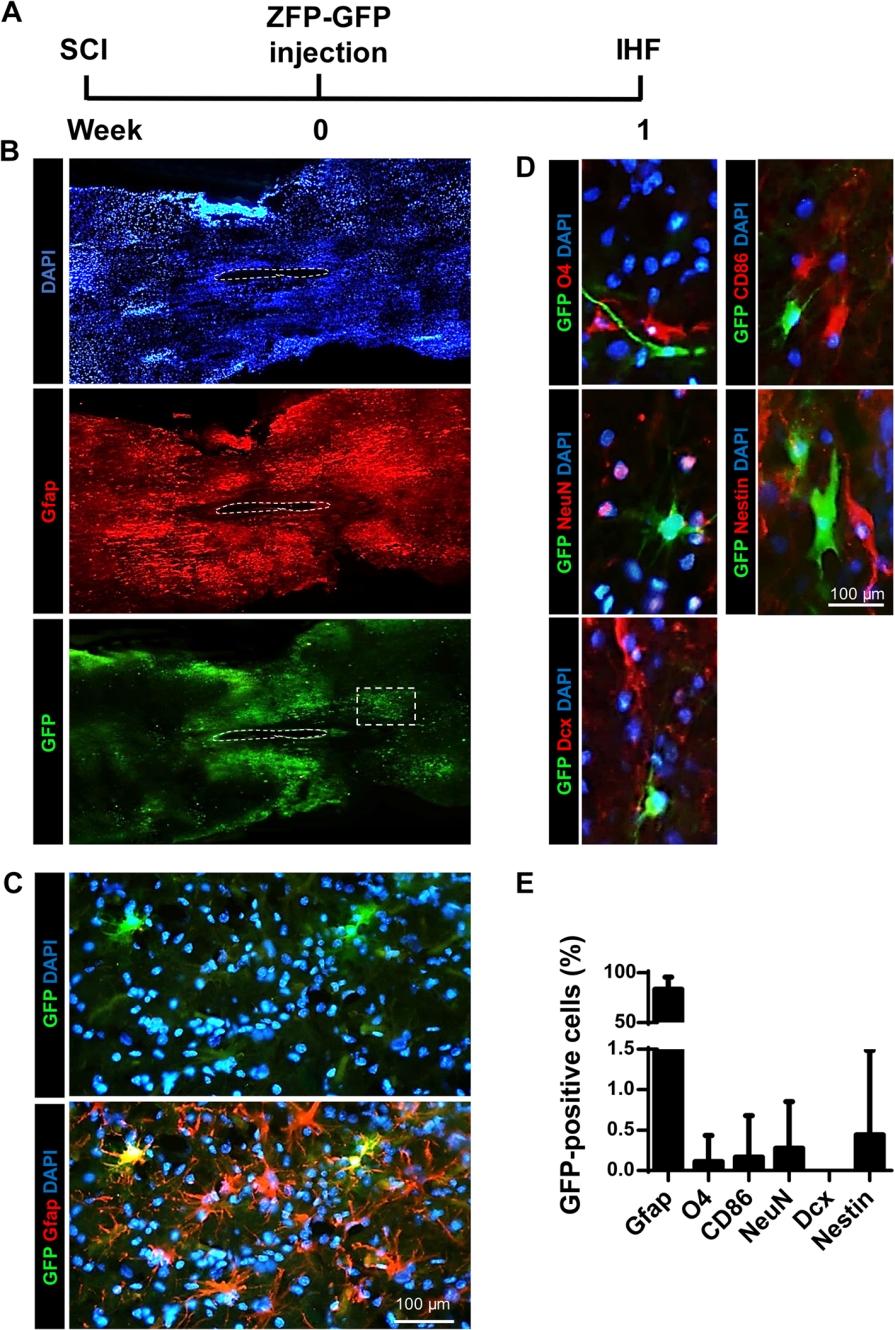
The targeted cells by the Zfp-GFP lentivirus. **a** Experimental design. SCI, spinal cord injury; IHF, immunohistofluorescence; W, Weeks. **b** GFP expression indicates transduced cells in the longitudinal sections of the spinal cord. Reactive astrocytes (AST) were stained using the glial fibrillary acidic protein (Gfap) antibody. The cavity in the injured site is outlined. Representative images showing GFP^+^ and Gfap^+^ cells in the spinal cord. Note that most of the GFP^+^ cells are detected around the glial scar in the site of injury (Gfap^+^ cells). **c** Higher magnification of insert in **b**. **d** Immunohistofluorescence analysis for GFP^+^ cells. Immunostaining was performed for Gfap (astrocytes), O4 (oligodendrocytes), CD86 (microglia), NeuN (neurons), Nestin (neural stem cells [NSCs]) and Dcx (neuroblast and immature neurons). **e** Quantification of protein markers revealed by immunostaining in **d**. Note that most of the GFP^+^ cells are Gfap^+^ (83.3%, reactive astrocytes). Data are presented as mean ± SD. Mean is shown for *n* = 3 animals. The number of counted cells is presented in Additional file 4: Table S3

We sought to identify possible mechanisms for the perceived functional improvement by examining the cell types targeted by the Zfp lentiviral system in our model. Immunohistofluorescence analysis of longitudinal sections indicated that GFP^+^ cells were present around the site of injection and scar at 1 WPT (Fig. 8a). The vast majority of GFP^+^ cells expressed the astrocyte-specific marker Gfap (83.3 ± 3.3%) (Fig. 8b, d). A small percentage of GFP^+^ cells stained positive for markers of oligodendrocyte precursors (O4), microglial cells (CD86), neurons (NeuN), and NSCs (Nestin) (Fig. 8c, d). We could not identify cells that co-expressed GFP and Dcx (neuroblasts) (Fig. 8c, d). These results demonstrated that our lentivirus primarily targeted spinal astrocytes under the regulation of the SFFV promoter.

**Fig. 9:**
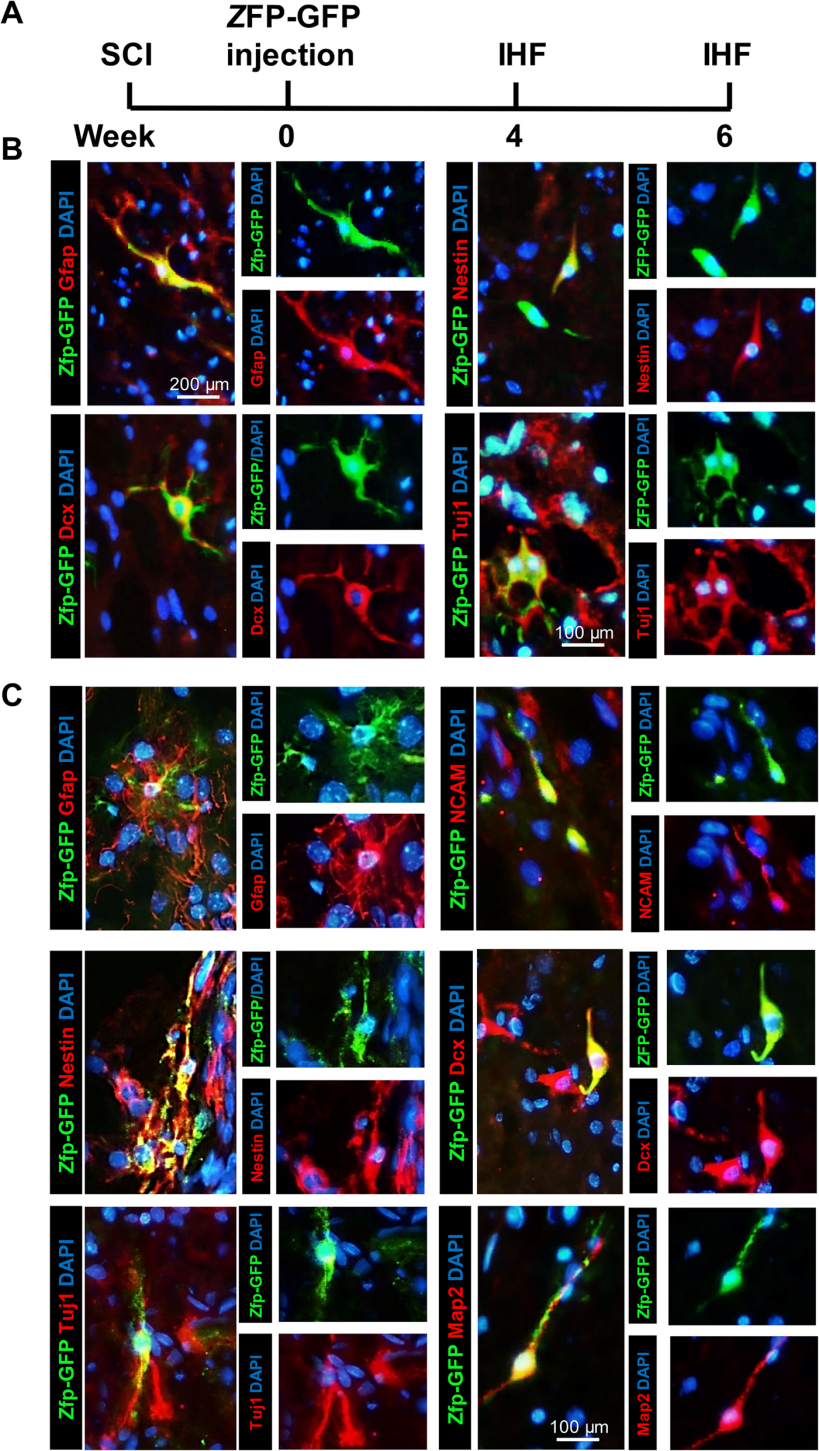
Neurogenesis induced by Zfp-GFP in the adult spinal cord at 4 and 6 weeks post-transduction (WPT). **a** Experimental design. SCI, spinal cord injury; IHF: immunohistofluorescence; W, weeks. **b** Representative images of immunofluorescence staining for spinal cord sections. Micrographs show merges of DAPI/GFP with Gfap (astrocytes), Nestin (neural stem cells), and β-tubulin III (Tuj1) after 4 weeks. **c** Representative images of immunofluorescence staining for spinal cord sections. Micrographs show merges of DAPI/GFP with Gfap, Nestin, Tuj1 and NCAM (immature neurons), Dcx (neuroblast and immature neurons), and MAP2 (mature neurons)

**Fig. 10:**
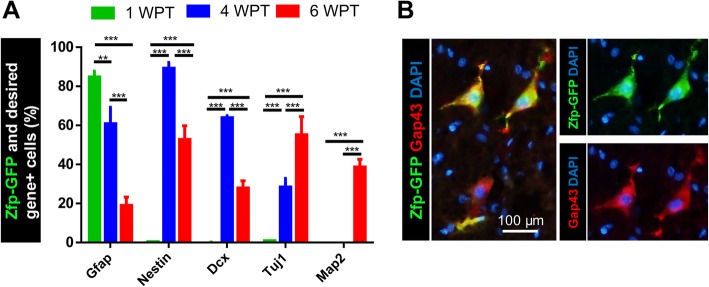
Quantitative analysis of immunofluorescence staining in the adult spinal cord. **a** Quantitative analysis of immunofluorescence staining. Data are represented as mean ± SD. Mean is shown for *n* = 3 animals in each group. The total cell number was 8149. Data were analyzed by ANOVA and Tukey test as post hoc. **p < 0.01 and ***p < 0.001. **b** Representative images of immunofluorescence staining for GAP43 in spinal cord sections at 6 weeks post-transduction (WPT). The number of counted cells is presented in Additional file 4: Table S3

We then examined Zfp-induced neurogenesis in the adult spinal cord using immunohistochemistry at 4 and 6 WPT (Fig. 9). At 4 WPT, the percentage of GFP and Gfap^+^ cells decreased and the percentage of GFP and Nestin^+^ or Dcx^+^ cells increased (Figs. 9a and 10a). However, at 6 WPT, the percentage of cells that co-expressed GFP and Nestin and Dcx decreased, and the percentage of Tuj1 and Map2 increased (Fig. 9b and 10a). These results are indicative of transdifferentiation of astrocytes into neurons.

Regenerating axons were shown by the expression of growth-associated protein 43 (Gap43), a protein that is expressed in growing axons during development and also after injury in adults. After 6 weeks, we observed Gap43^+^ and GFP^+^ cells around the scar areas (Fig. 10b).

**Fig. 11:**
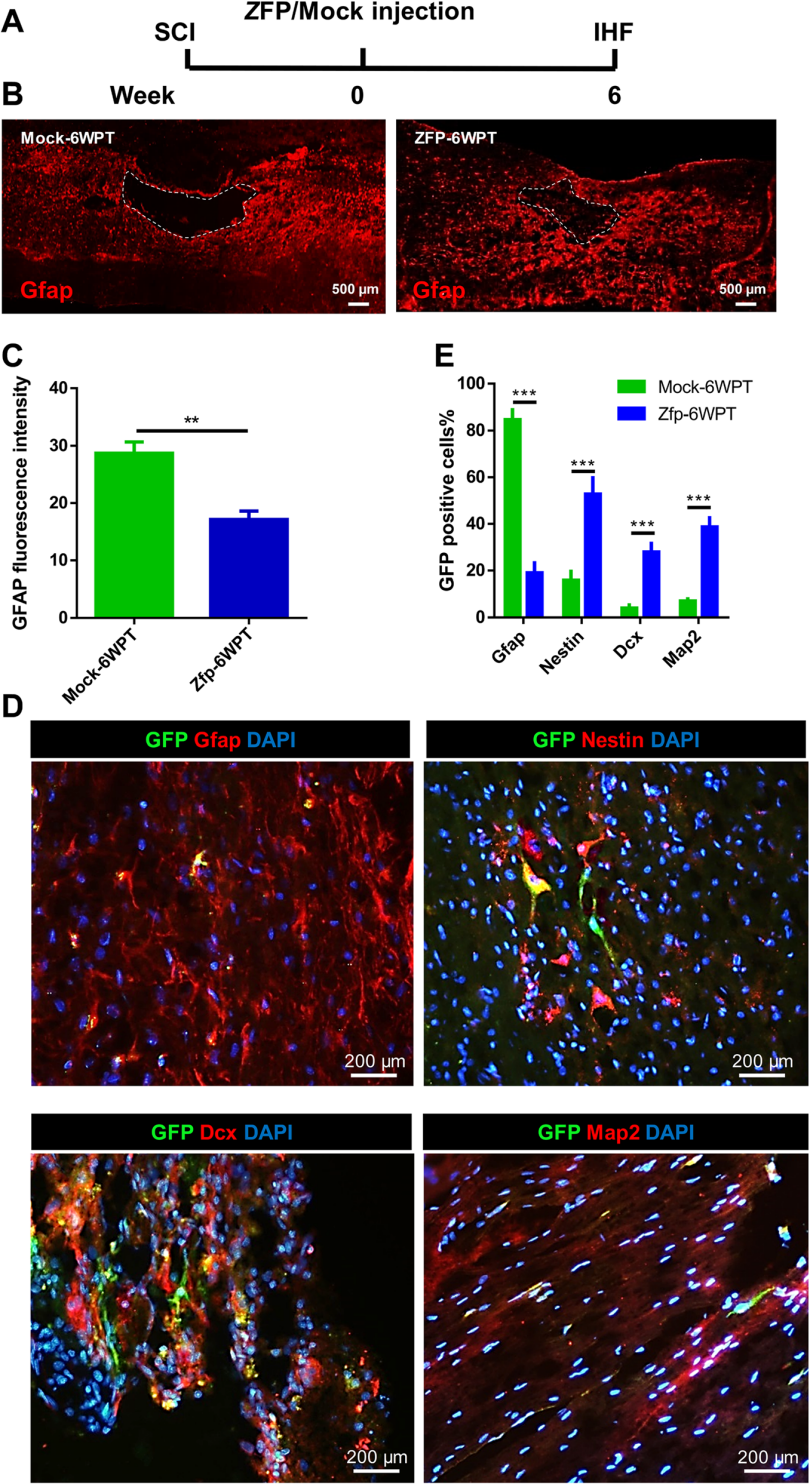
The comparison of the cell fate of induced neural stem cells (iNSC) in the Mock and Zfp-GFP groups at 6 weeks post-transduction (WPT). **a** Experimental design. SCI, spinal cord injury; IHF, immunohistofluorescence; W, weeks. **b** Representative images of Gfap staining for spinal cord sections in the Mock and Zfp groups. **c** Immunostaining of the spinal cord in the Mock group at 6 WPT. Micrographs showing merges of DAPI/GFP with Gfap (astrocytes), Nestin (neural stem cells), Dcx (neuroblasts), and Map2 (mature neurons) after 6 weeks. **d** Fluorescence intensity after immunostaining for Gfap. **e** Quantitative analysis of immunofluorescence staining for the Mock and Zfp group. Mean is shown for *n* = 3 animals in each group. Data are represented mean ± SD. Data were analyzed by the unpaired *t* test. **p < 0.01; ***p < 0.001. The number of counted cells is presented in Additional file 4: Table S3

Assessment of the spinal cord sections in the Mock and Zfp groups at 6 WPT (Fig. 11a) showed the cavity area, and there was significantly reduced Gfap fluorescence intensity in the Zfp group (Fig. 11b, c). The majority of GFP^+^ cells in the Mock group were Gfap^+^ astrocytes, whereas the majority of GFP^+^ cells in the Zfp group were Nestin^+^ and Map2^+^ cells (Fig. 11d, e).

## Discussion

We have shown that rat astrocytes in culture can be reprogrammed into iNSCs by overexpression of *Zfp521.* Interestingly, the reprogramming efficiency of this transcription factor in vitro is more than 40% in rat astrocytes but only 0.2% in fibroblasts [31]*.* This difference in reprogramming efficiency may be related to the lineage relationship between NSCs and glial cells. We selected *Zfp521* for in vivo astrocyte to neuron transdifferentiation because this transcription factor directly activates early neural genes such as *Sox3*, *Sox1*, and *Pax6* [42]*.* In addition, *Zfp521* is a key factor in inducing differentiation of mouse embryonic stem cells into neural progenitors and its knockdown leads to inhibition of neural differentiation [42]. *Zfp521* has been shown to be sufficient for the direct conversion of cultured human and mouse fibroblasts [31] as well as mouse astrocytes [32] into NSCs. Moreover, in comparison with *Sox2* overexpression, *Zfp521* demonstrated a higher efficiency in the conversion of astrocytes into iNSCs. There was no increase in apoptosis and senescence with passaging of iNSCs. These iNSCs had the potential to differentiate into neurons, astrocytes, and oligodendrocytes.

Our results showed that after the SCI, rat spinal cord astrocytes could be indirectly reprogrammed into functional neurons in vivo through a progenitor stage using a single transcription factor, *Zfp521*. *Zfp521*-mediated reprogramming is a slow process with neuroblasts or mature neurons not readily detectable before 4 WPT. Reprogramming glial cells into neurons after a SCI could potentially constitute a therapeutic approach for treating reactive gliosis, which is widely associated with nerve injury.

This study demonstrated that *Zfp521* could generate mature neurons gradually through the generation of Nestin^+^ and Dcx^+^ cells which could, in turn, improve functional abilities of the rats as detected by the BBB scale of locomotor recovery and further shown by calculation of the subscores. The locomotor activity of the hind limbs, certain footprint parameters, step length, toe spread, foot length, and paw area improved in the *Zfp521* group. The MEP data suggest potential integration of reprogrammed cells into the local neural network of the injured spinal cord and the formation of intraspinal relays that are able to mediate communication between segments above and below the lesion site.

Following an injury to the central nervous system, astrocytes become activated and show stem cell properties as they proliferate and form neurospheres in vitro [43, 44]. However, these activated astrocytes cannot spontaneously produce neurons in vivo [43, 44]. Our forced expression of *Zfp521* supports the astrocytes to enable them to overcome epigenetic barriers and convert into NSCs.

We could not detect any newly generated neurons in the injured spinal cords that were injected with lentivirus expressing the control GFP under the SFFV promoter. Genetic lineage tracings showed that ectopic Zfp521 uniquely converted resident astrocytes into Nestin^+^ and Dcx^+^ neuroblasts and Map2^+^ mature neurons in the adult spinal cord. Endogenous stem cells are also amenable to fate programming, and Zfp-GFP rarely expressed Nestin^+^ or Dcx^+^ cells at 1 WPT. In addition, we observed new axon growth by the growth-associated protein Gap43. This protein is an axonal phosphoprotein that is expressed at high levels during development and regeneration. Consequently, studies have shown that Gap43 is a key molecule in the regulation of axonal growth. The presence of GFP/Gap43-labeled axons after injury and in the Zfp group indicated that axonal growth began in astrocyte-derived neurons at 6 WPT. It has been shown that ectopic expression of the transcription factor *NeuroD1* or *Sox2* is sufficient to directly convert brain or spinal cord astrocytes into neurons [21]. It was also reported that forced expression of *CenD1*, *NeuroG2*, or the combination of both resulted in transdifferentiation of brain astrocytes into iNSCs and neurons [30]. It has been reported that the fate of resident astrocytes can be altered to become neuroblasts and mature neurons in the adult spinal cord [29] or brain [20] through ectopic *Sox2* expression or miR-302/367 in combination with valproic acid treatment [45].

## Conclusion

The results of our study suggested a potential role for *Zfp521* to improve recovery following SCI by reprogramming endogenous astrocytes that are recruited to the site of injury. This therapeutic approach will likely face fewer hurdles in the clinic since exogenous cell transplantation is not required. However, the reprogramming efficiency of this transcription factor is low and further studies should look into improving the number of converted neurons. Furthermore, protocols should be described to yield subtype-specific neurons as is required for functional recovery following SCI. The results of this study provide new insights into the cellular processes of *Zfp521*-driven in vivo reprogramming, which may be useful for future strategies aimed at improving reprogramming efficiency and the derivation of disease-relevant neuron subtypes. In addition, the reprogramming of human astrocytes into functional neurons suggests that such a reactive glia-neuron conversion approach is potentially applicable to human patients.

## Supplementary information

**Additional file 1: Figure S1.** Isolation and characterization of adult rat brain astrocytes. (A) Phase-contrast images of astrocytes. (B) RT-PCR analysis of astrocytes and neural stem cell (NSC) markers. (C) qRT-PCR analysis of astrocytes and NSC markers. Data were normalized against *GAPDH* and presented relative to the expression of each indicated gene in the astrocytes. (D) Immunostaining images for astrocyte markers. Nuclei were counterstained with DAPI. (E) Quantification of the immunostainings for astrocytes and NSC markers. Data in C and E are shown as mean ± SD of three biological replicates. ND: Not detected. The number of counted cells is presented in Additional file 4: Table S3.

**Additional file 2: Figure S2.** The validation of inducible vector. (A) Immunostaining for astrocyte and neural stem cell (NSC) markers in transfected astrocytes. Transduction was performed with an empty vector under two conditions: Astrocytes in astrocyte medium without DOX (A) and astrocytes in induction medium (IM) and DOX (AD). In addition, astrocytes were transfected by the *Zfp521* vector in the present and the absence of Dox in IM (AZD and AZ, respectively). Nuclei were counterstained with DAPI. (B) Quantification of the immunostainings for astrocytes and NSC markers in transfected astrocytes. Data are shown as mean ± SD of three biological replicates. Data were analyzed by ANOVA and Mann-Whitney U test as post hoc. ***: *p* < 0.001. The number of counted cells is presented in Additional file 4: Table S3.

**Additional file 3: Figure S3.** Morphology and histology of the spinal cord injury (SCI) in s contusion model. (A) The adult male Wistar rat’s spinal cord was exposed at T10. Through a dorsal midline incision, a T9–11 laminectomy was performed and the spinal cord was exposed. A contusion injury was made using a standardized weight-drop injury device (NYU impactor) as indicated by hemorrhage. (B) Hematoxylin and eosin (H&E) staining of the rat injured spinal cord sections at one week post-injury. The lesion core and glial scar are shown in the longitudinal section. (C) Transverse images show Gfap-immunoreactive astrocytes at the injured, caudal, and rostral sites. Note that the expression of Gfap was higher around the injured site relative to the caudal and rostral sites. (D) Double immunostaining for fibronectin (green) and Gfap (red) in a longitudinal spinal cord section one week post-injury in the rat injured spinal cord. The lesion site was filled with a fibronectin positive matrix (fibrotic scar or lesion core) and surrounded with a Gfap^+^ area (glial scar).

**Additional file 4: Table S1.** List of antibodies used for immunostaining. **Table S2.**Primer sequences used for quantitative real-time PCR (qRT-PCR). **Table S3.** The number of cells in each immunostaining analysis.

## Data Availability

All data generated or analyzed during this study are included in this published article [and its supplementary information files].
