# Supplementary figures and images for: In vivo conversion of rat astrocytes into neuronal cells through neural stem cells in injured spinal cord with a single zinc-finger transcription factor
Source: Stem Cell Res Ther. 2019 Dec 16;10:380. doi: 10.1186/s13287-019-1448-x (PMC6916443; doi:10.1186/s13287-019-1448-x)

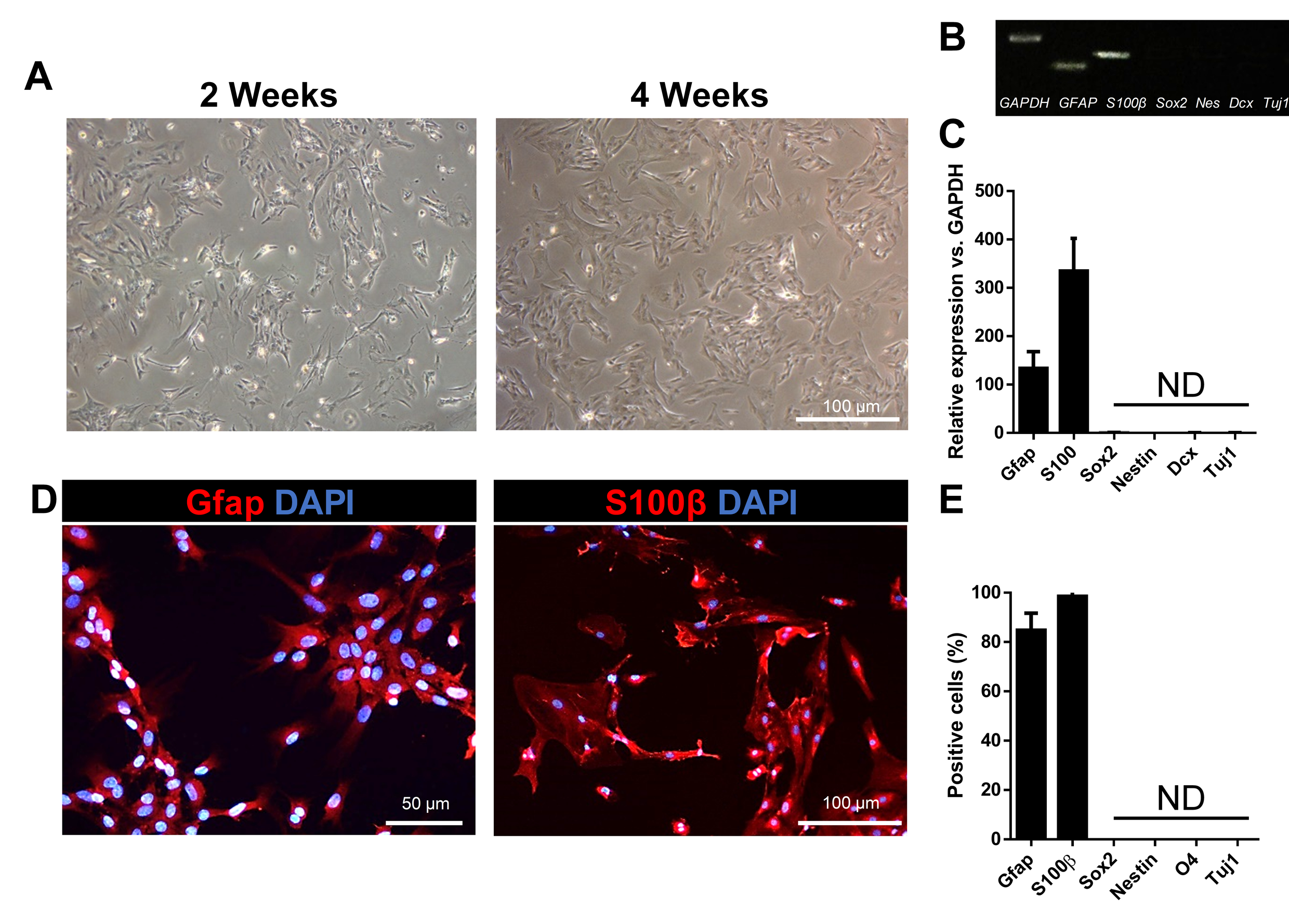

Supplement: Supplementary file 1 — Additional file 1: Figure S1. Isolation and characterization of adult rat brain astrocytes. (A) Phase-contrast images of astrocytes. (B) RT-PCR analysis of astrocytes and neural stem cell (NSC) markers. (C) qRT-PCR analysis of astrocytes and NSC markers. Data were normalized against GAPDH and presented relative to the expression of each indicated gene in the astrocytes. (D) Immunostaining images for astrocyte markers. Nuclei were counterstained with DAPI. (E) Quantification of the immunostainings for astrocytes and NSC markers. Data in C and E are shown as mean ± SD of three biological replicates. ND: Not detected. The number of counted cells is presented in Additional file 4: Table S3. [file 13287_2019_1448_MOESM1_ESM.tif]

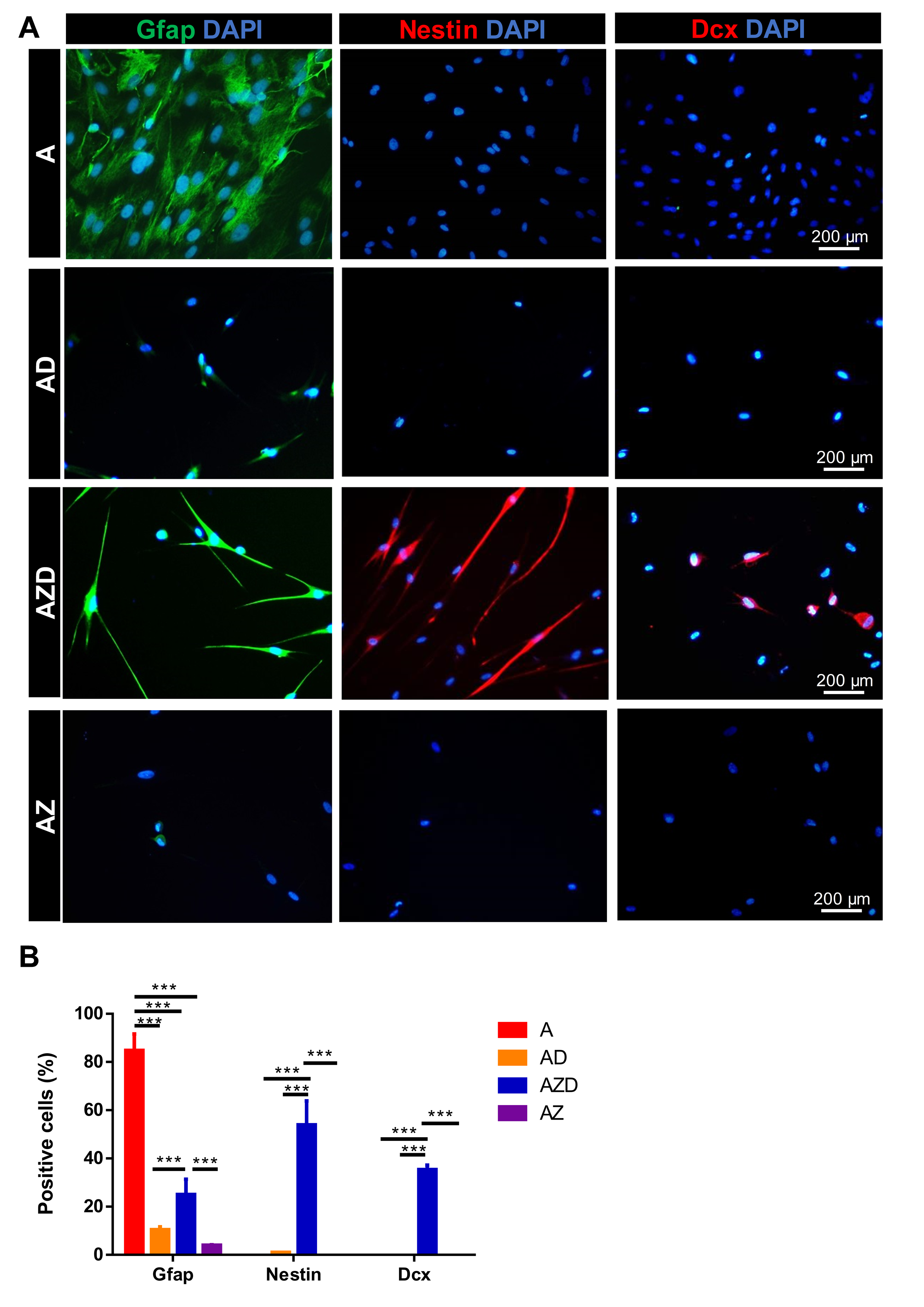

Supplement: Supplementary file 2 — Additional file 2: Figure S2. The validation of inducible vector. (A) Immunostaining for astrocyte and neural stem cell (NSC) markers in transfected astrocytes. Transduction was performed with an empty vector under two conditions: Astrocytes in astrocyte medium without DOX (A) and astrocytes in induction medium (IM) and DOX (AD). In addition, astrocytes were transfected by the Zfp521 vector in the present and the absence of Dox in IM (AZD and AZ, respectively). Nuclei were counterstained with DAPI. (B) Quantification of the immunostainings for astrocytes and NSC markers in transfected astrocytes. Data are shown as mean ± SD of three biological replicates. Data were analyzed by ANOVA and Mann-Whitney U test as post hoc. ***: p < 0.001. The number of counted cells is presented in Additional file 4: Table S3. [file 13287_2019_1448_MOESM2_ESM.tif]

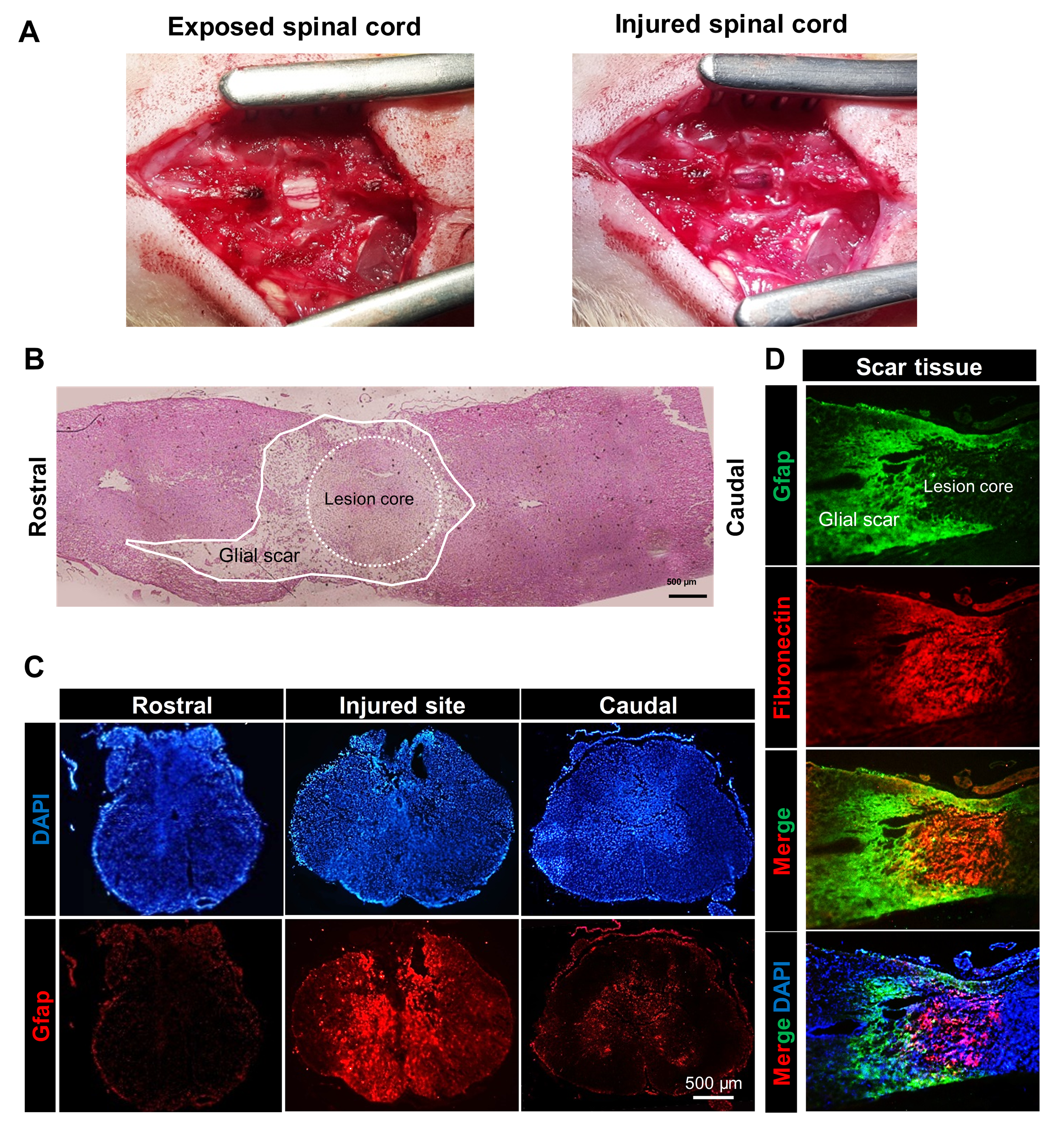

Supplement: Supplementary file 3 — Additional file 3: Figure S3. Morphology and histology of the spinal cord injury (SCI) in s contusion model. (A) The adult male Wistar rat’s spinal cord was exposed at T10. Through a dorsal midline incision, a T9–11 laminectomy was performed and the spinal cord was exposed. A contusion injury was made using a standardized weight-drop injury device (NYU impactor) as indicated by hemorrhage. (B) Hematoxylin and eosin (H&E) staining of the rat injured spinal cord sections at one week post-injury. The lesion core and glial scar are shown in the longitudinal section. (C) Transverse images show Gfap-immunoreactive astrocytes at the injured, caudal, and rostral sites. Note that the expression of Gfap was higher around the injured site relative to the caudal and rostral sites. (D) Double immunostaining for fibronectin (green) and Gfap (red) in a longitudinal spinal cord section one week post-injury in the rat injured spinal cord. The lesion site was filled with a fibronectin positive matrix (fibrotic scar or lesion core) and surrounded with a Gfap+ area (glial scar). [file 13287_2019_1448_MOESM3_ESM.tif]
