# Supplementary material for: In vivo conversion of rat astrocytes into neuronal cells through neural stem cells in injured spinal cord with a single zinc-finger transcription factor
Source: Stem Cell Res Ther. 2019 Dec 16;10:380. doi: 10.1186/s13287-019-1448-x (PMC6916443; doi:10.1186/s13287-019-1448-x)
Supplement: Supplementary file 4 — Additional file 4: Table S1. List of antibodies used for immunostaining. Table S2.Primer sequences used for quantitative real-time PCR (qRT-PCR). Table S3. The number of cells in each immunostaining analysis. [file 13287_2019_1448_MOESM4_ESM.docx]

**Table S1.** List of antibodies used for immunostaining.

| Primary antibody | Company | Cat # | Concentration |
| --- | --- | --- | --- |
| Nestin | Sigma-Aldrich | SAB4200347 | 1:200 |
| S100β | Sigma-Aldrich | S2644 | 1:200 |
| Map2 | Sigma-Aldrich | M1406 | 1:200 |
| Tuj1 | Sigma-Aldrich | T8328 | 1:200 |
| NF200 | Sigma-Aldrich | N0142 | 1:200 |
| Gfap | Sigma-Aldrich | G3893 | 1:200 |
| O4 | Sigma-Aldrich | O7139 | 1:200 |
| Olig2 | Sigma-Aldrich | Q13516 | 1:200 |
| Sox1 | Abcam | Ab22572 | 1:200 |
| Dcx | Abcam | Ab23544-100 | 1:200 |
| NCAM | Abcam | ab6123 | 1:200 |
| NeuN | Abcam | ab104224 | 1:200 |
| CD86 | Abcam | ab220188 | 1:200 |
| Gaba-A receptor | Abcam | ab33299 | 1:200 |
| Caspase 3 | Santa Cruz | sc-56052 | 1:200 |
| Annexin V | Santa Cruz | sc-74438 | 1:200 |
| Gap43 | Invitrogen | 33-5000 | 1:200 |
| Secondary antibody |  |  |  |
| Goat anti mouse IgG | Invitrogen | A11004 | 1/1000 |
| Donkey anti rabbit IgG | Invitrogen | A10040 | 1/1000 |

**Table S2.** Primer sequences used for quantitative real-time PCR (qRT-PCR).

| Gene name | Forward primer (5'-3') | Reverse primer (5'-3') |
| --- | --- | --- |
| *GAPDH* | TGCTGAGTATGTCG GGAGTC | AAAGGTGGAAGAATGGGAG |
| *Gfap* | AGTGGTATCGGTCCAAGTTTGC | TGGCGGCGATAGTCATTAGC |
| *S100β* | TCACTGAGGGACGAAATCAACAC | GGTGCTATTGGTAGTCTGCCTTG |
| *Sox2* | GCTGGGAGAAAGAAGAGGAG | ATCTGGCGGAGAATAGTTGG |
| *Nestin* | AACCACAGGAGTGGGAACTG | TCTGGCATTGACTGAGCAAC |
| *Zp521* exogene | ACAGCTCCGAGCCTATGTACG | GGTCTGATGTTGTGGTCCCTC |
| *Zfp521* endogene | CTCAGTGGACAGCTCAACCA | ACCGCGAGACTCGAGAATAA |
| *Dcx* | GGGGATTGTGTACGCTGTTT | CGACCAGTTGGGATTGACAT |
| *Tuj1* | TGAGGCCTCCTCTCACAAGT | TGCAGGCAGTCACAATTCTC |

**Table S3.** The number of cells in each immunostaining analysis.

| **Figure 1** | Gfap | S100β | Sox1 | Nestin | Dcx |
| --- | --- | --- | --- | --- | --- |
| AST | 1159 | 2189 | 1092 | 982 | 2670 |
| Zfp | 774 | 386 | 461 | 647 | 2402 |
| Sox2 | 828 | 1146 | 668 | 969 | 1579 |
| 2TF | 797 | 611 | 561 | 1081 | 1630 |

| **Figure 2** | Gfap | S100β | Zfp521 | Sox1 | Pax6 | Nestin | Dcx | Tuj1 |
| --- | --- | --- | --- | --- | --- | --- | --- | --- |
| AST | 1159 | 2210 | 879 | 479 | 908 | 498 | 689 | 549 |
| P5 | 1251 | 1287 | 485 | 849 | 657 | 1028 | 1462 | 900 |

| **Figure 3** | Caspase 3 | Annexin V |
| --- | --- | --- |
| Number | 3871 | 3883 |

| **Figure 8** | Gfap | Nestin | Dcx | NeuN | CD86 | O4 |
| --- | --- | --- | --- | --- | --- | --- |
| Number | 1077 | 340 | 254 | 975 | 238 | 692 |

| **Figure 10** | Gfap | Nestin | Dcx | Tuj1 | Map2 |
| --- | --- | --- | --- | --- | --- |
| 2 WPT | 1077 | 340 | 596 | 479 | 439 |
| 4 WPT | 746 | 472 | 460 | 545 | 598 |
| 6 WPT | 374 | 345 | 420 | 849 | 409 |

| **Figure 11** | Gfap | Nestin | Dcx | Map2 |
| --- | --- | --- | --- | --- |
| Mock-6 WPT | 342 | 238 | 290 | 355 |
| ZFP-6 WPT | 374 | 345 | 420 | 409 |

| **Figure S1** | Gfap | S100β | Sox2 | Nestin | Tuj1 | O4 |
| --- | --- | --- | --- | --- | --- | --- |
| Number | 1120 | 2210 | 840 | 1120 | 549 | 903 |

| **Figure S2** | Gfap | Nestin | Dcx |
| --- | --- | --- | --- |
| Number | 2450 | 2479 | 1279 |
